# Risk factors for maternal death and trends in maternal mortality in low- and middle-income countries: a prospective longitudinal cohort analysis

**DOI:** 10.1186/1742-4755-12-S2-S5

**Published:** 2015-06-08

**Authors:** Melissa Bauserman, Adrien Lokangaka, Vanessa Thorsten, Antoinette Tshefu, Shivaprasad S Goudar, Fabian Esamai, Ana Garces, Sarah Saleem, Omrana Pasha, Archana Patel, Albert Manasyan, Mabel Berrueta, Bhala Kodkany, Elwyn Chomba, Edward A Liechty, K Michael Hambidge, Nancy F Krebs, Richard J Derman, Patricia L Hibberd, Fernando Althabe, Waldemar A Carlo, Marion Koso-Thomas, Robert L Goldenberg, Dennis D Wallace, Elizabeth M McClure, Carl L Bose

**Affiliations:** 1 Department of Pediatrics, Division of Neonatal-Perinatal Medicine, University of North Carolina School of Medicine, Chapel Hill, NC, USA; 2 Kinshasa School of Public Health, Kinshasa, Democratic Republic of Congo; 3 RTI International, Durham, NC, USA; 4 KLE University’s Jawaharlal Nehru Medical College, Belgaum, India; 5 Moi University School of Medicine, Eldoret, Kenya; 6 Fundación para la Alimentación y Nutrición de Centro América y Panamá, Guatemala City, Guatemala; 7 Aga Khan University, Karachi, Pakistan; 8 Lata Medical Research Foundation, Nagpur, India; 9 University Teaching Hospital, Lusaka, Zambia; 10 Institute for Clinical Effectiveness and Health Policy, University of Buenos Aires, Argentina; 11 School of Medicine, University of Indiana, Indianapolis, IN, USA; 12 University of Colorado School of Medicine, Colorado, USA; 13 Christiana Health Care, Newark, DE, USA; 14 Massachusetts General Hospital, Boston, MA, USA; 15 University of Alabama at Birmingham, Birmingham, AL, USA; 16 Eunice Kennedy Shriver National Institute of Child Health and Human Development, Bethesda, MD, USA; 17 Department of Obstetrics and Gynecology, Columbia University School of Medicine, USA

## Abstract

**Background:**

Because large, prospective, population-based data sets describing maternal outcomes are typically not available in low- and middle-income countries, it is difficult to monitor maternal mortality rates over time and to identify factors associated with maternal mortality. Early identification of risk factors is essential to develop comprehensive intervention strategies preventing pregnancy-related complications. Our objective was to describe maternal mortality rates in a large, multi-country dataset and to determine maternal, pregnancy-related, delivery and postpartum characteristics that are associated with maternal mortality.

**Methods:**

We collected data describing all pregnancies from 2010 to 2013 among women enrolled in the multi-national Global Network for Women’s and Children’s Health Research Maternal and Neonatal Health Registry (MNHR). We reported the proportion of mothers who died per pregnancy and the maternal mortality ratio (MMR). Generalized linear models were used to evaluate the relationship of potential medical and social factors and maternal mortality and to develop point and interval estimates of relative risk associated with these factors. Generalized estimating equations were used to account for the correlation of outcomes within cluster to develop appropriate confidence intervals.

**Results:**

We recorded 277,736 pregnancies and 402 maternal deaths for an MMR of 153/100,000 live births. We observed an improvement in the total MMR from 166 in 2010 to 126 in 2013. The MMR in Latin American sites (91) was lower than the MMR in Asian (178) and African sites (125). When adjusted for study site and the other variables, no formal education (RR 3.2 [1.5, 6.9]), primary education only (RR 3.4 [1.6, 7.5]), secondary education only (RR 2.5 [1.1, 5.7]), lack of antenatal care (RR 1.8 [1.2, 2.5]), caesarean section delivery (RR 1.9 [1.3, 2.8]), hemorrhage (RR 3.3 [2.2, 5.1]), and hypertensive disorders (RR 7.4 [5.2, 10.4]) were associated with higher risks of death.

**Conclusions:**

The MNHR identified preventable causes of maternal mortality in diverse settings in low- and middle-income countries. The MNHR can be used to monitor public health strategies and determine their association with reducing maternal mortality.

**Trial Registration:**

clinicaltrials.gov NCT01073475

## Background

More than 99% of the women who die from pregnancy-related complications live in low- and middle-income countries where systematic data collection is of variable quality [1-4]. In these countries, most data sets are incomplete or record small numbers of deaths. Because maternal deaths occur infrequently, it is difficult to draw inferences about the underlying causes of mortality from these data sets. In addition to the relatively small numbers of maternal deaths, many reports are limited to women who were treated in facilities, which might underrepresent mortality due to deaths that occur at home or outside of a health care facility. Also, they misclassify maternal death as that being attributable to causes other than pregnancy-related causes [5-8]. Because prospective data sets that record large numbers of maternal deaths are lacking, much of the information available relative to maternal mortality is derived from models that generate estimates or synthesize data from several studies or data repositories [5,9,10]. Therefore, a reliable, comprehensive set of medical and social factors that are strongly associated with maternal mortality is needed from prospective population-based data sets that can also track maternal mortality rates over time. Early identification of risk factors is essential to develop comprehensive intervention strategies preventing pregnancy-related complications. Maternal mortality rates will not be reduced without comprehensive intervention strategies that include monitoring of risk factors shown to be associated with maternal mortality [11,12].

The *Eunice Kennedy Shriver* National Institute of Child Health and Human Development (NICHD) Global Network for Women’s and Children’s Health Research (GN) Maternal and Neonatal Health Registry (MNHR) is a five-year, multi-country, population-based, prospectively collected record of pregnancy outcomes including maternal death. This data set is ideal to study a variety of medical and social risk factors that might contribute to maternal mortality. The goals of this paper are to describe maternal mortality rates by site within the GN from 2010 to 2013 and to determine maternal, pregnancy-related, delivery and postpartum characteristics that are associated with maternal mortality.

## Methods

We collected information from the MNHR for pregnancies between January 1, 2010 and December 31, 2013. The registry included research sites in the counties of Busia, Bungoma and Kakamega (within the western region), Kenya; Kafue and Chongwe (located south and east of the capital city of Lusaka), Zambia; Belgaum and Bagalkot (within the northern part of the southern state of Karnataka), India; Nagpur (within the state of Mahrashtra), India; Thatta (two of the five sub-districts in the southern Sindh province, near the city of Karachi), Pakistan; Corrientes and Santiago del Estero, Argentina; and Chimaltenango (in the Western Highlands), Guatemala. The sites represent urban and rural environments across a range of human development indices (a composite score statistic of life expectancy, education and income) from 0.465-0.828.

Data were collected from women who resided within study clusters through a combination of methods, including abstraction of medical records and by a series of interviews conducted by trained study staff. Maternal characteristics, including demographic information were collected at the time a pregnant woman was screened and consented. Although the goal was to identify women as early as possible in pregnancy, women could be enrolled at any point during pregnancy or after delivery. Antenatal and delivery characteristics were recorded within 3-7 days after delivery. Postpartum characteristics were collected at a clinic or home visit at 6 weeks after delivery. This information was collected regardless of maternal or infant status at 6 weeks after delivery. We excluded women who were lost to follow-up prior to delivery and those who did not have records indicating maternal status at the 6-week follow-up visit. Pregnancy outcomes, including stillbirths and neonatal characteristics, have been previously published and are therefore not included in this paper [13].

We defined maternal death as the death of a woman while pregnant or within 42 days of the end of pregnancy, in accordance with the World Health Organization (WHO). We defined fetal malposition as transverse lie, oblique lie or breech. For maternal, antenatal, delivery and post-partum characteristics, we reported the percent of births resulting in a maternal death, using all recorded pregnancies as the denominator irrespective of the delivery outcome (miscarriage, stillbirth, live birth, etc). We also used the standard maternal mortality ratio (MMR=maternal deaths/100,000 live births) to record maternal death by site over time. The 95% confidence interval for the maternal mortality ratio was approximated using the variance of the proportion of maternal deaths for each site and year.

Generalized linear models were used to evaluate the relationship of potential medical and social factors and maternal mortality and to develop point and interval estimates of relative risk associated with these factors. Generalized estimating equations were used to account for the correlation of outcomes within cluster to develop appropriate confidence intervals. We ran a multivariable regression model to determine the maternal, pregnancy-related, delivery and postpartum factors that were associated with maternal death. We included all medical and social variables that could be associated with maternal mortality and were reliably collected in the MNHR. We included variables that were present at enrollment or around the time of delivery, including: maternal age, maternal education, parity, antenatal care, birth attendant, delivery mode, obstructed labor, fetal malposition, hemorrhage and hypertensive disorders. We excluded factors with significant missing fields. For the purposes of the multivariable model, we defined the variable hemorrhage as an antepartum or postpartum hemorrhage, and the variable hypertensive disorder as an antepartum hypertensive disorder or postpartum seizure or convulsion. The model was further adjusted for clinical site. Data are presented as adjusted risk ratios and 95% confidence intervals. All analyses were performed at the Data Coordinating Center at RTI International (Durham, NC) using SAS, Inc. (Version 9.3).

At each site, institutional review boards or research ethics committees and ministries of health approved the collection of data included in the MNHR. We used sensitization meetings to achieve approval within local communities prior to the initiation of the study, with informed consent from individual participants. A data monitoring committee, appointed by the NICHD oversees and reviews the MNHR annually.

## Results

**Figure 1:**
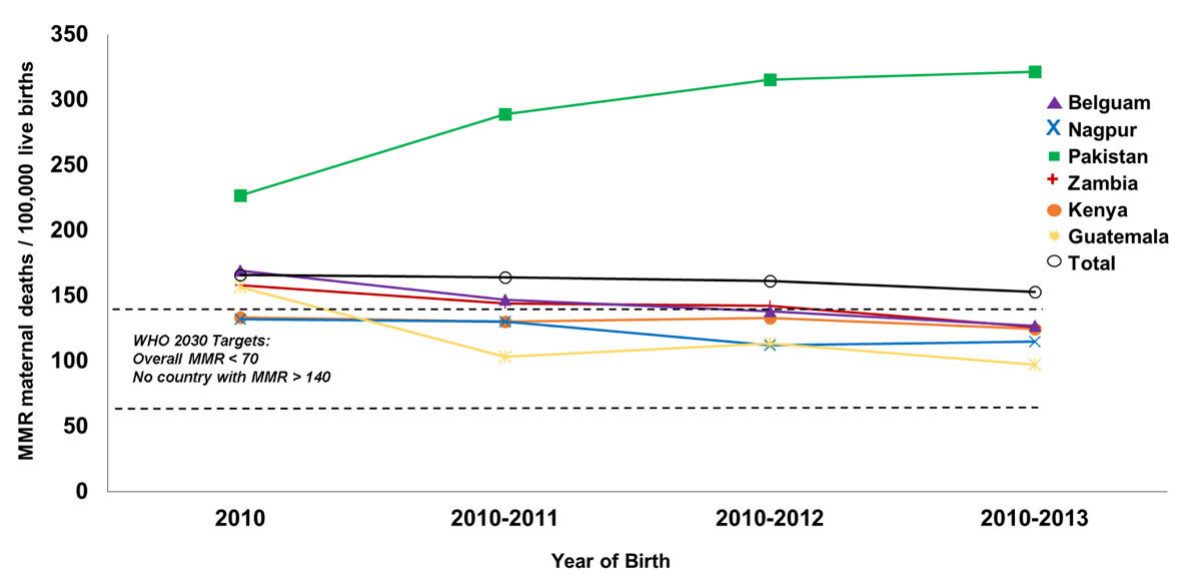
Cumulative maternal mortality ratio by site and years^*^ Individual site data for Argentina excluded due to incomplete data for year 2013. Data from Argentina are included in the overall total. ^*^ MNHR 2010-2013 deliveries excluding women lost to follow-up prior to delivery or missing maternal status at 6 weeks after delivery.

During the study period, we recorded 277,736 deliveries, 262,887 live births and 402 maternal deaths (145 maternal deaths/100,000 deliveries). This resulted in a MMR of 153/100,000 live births. We calculated maternal mortality ratios by site (Table 1). MMR ranged from 72 in Argentina to 321 in Pakistan. The MMR in Latin American sites (91) was lower than the MMR in Asian (178) and African sites (125). We observed an overall decrease in the total MMR over a four-year period from 166 in 2010 to 126 in 2013. Sites that had reductions in the MMR in the four-year period included: Kenya (133 to 98), Zambia (158 to 76), Belgaum, India (169 to 87), Nagpur, India (132 to 123), and Guatemala (157 to 68). Pakistan had the highest MMR each year compared to the other sites and the MMR increased from 2010 to 2013 (227 to 348). Cumulative maternal mortality is illustrated in Figure 1.

**Table 1:** Maternal mortality ratio (MMR) by site and year

Region	Africa		Asia			Latin America		Total
		
Site	Kenya	Zambia	Belgaum, India	Nagpur, India	Pakistan	Argentina	Guatemala	
Births, n	35,603	27,614	84,573	41,293	49,602	9,860	29,191	277,736

Maternal deaths, n	44	34	99	44	146	7	28	402

Deliveries, n	36,060	27,863	85,160	41,574	50,191	9,921	29,392	280,161

Live births, n	35,300	26,997	78,189	38,326	45,439	9,781	28,855	262,887

Total MMR	124.6	125.9	126.6	114.8	321.3	71.6	97.0	152.9

MMR, (95% CI) ^‡^								

2010	133.3 (56.9, 209.7)	157.8 (63.9, 251.7)	169.2 (112.1, 226.4)	132.1 (59.8, 204.3)	226.6 (146.2, 306.9)	106.6 (0.0, 228.1)	156.5 (47.6, 265.5)	165.9 (135.0, 196.8)

2011	127.1 (54.5, 199.7)	130.1 (44.5, 215.6)	124.0 (75.2, 172.9)	128.1 (55.4, 200.8)	354.7 (251.3, 458.1)	99.2 (0.0, 212.3)	61.1 (0.8, 121.4)	162.2 (131.8, 192.6)

2012	138.1 (59.2, 217.1)	137.4 (47.1, 227.6)	120.6 (73.0, 168.1)	74.9 (19.1, 130.6)	382.9 (263.3, 502.5)	0.0 (0.0, 0.0)	131.3 (45.1, 217.5)	155.5 (125.1, 185.9)

2013	98.0 (35.6, 160.3)	76.3 (13.0, 139.6)	87.2 (49.2, 125.1)	122.9 (53.5, 192.3)	347.7 (270.0, 425.5)	*	67.7 (0.0, 169.6)	125.5 (99.9, 151.2)

Total	124.6 (87.4, 161.9)	125.9 (83.3, 168.6)	126.6 (101.5, 151.7)	114.8 (80.7, 148.9)	321.3 (268.8, 373.8)	71.6 (18.3,124.9)	97.0 (60.9, 133.2)	152.9 (137.9, 168.0)

^*^ Complete data are not available for Argentina for year 2013

^‡^ The 95% confidence interval for the MMR approximated using the variance of the proportion of maternal deaths for each site and year.

Mothers who were >35 years old, had no formal education or primary education only, had >2 children, or had a previous pregnancy loss were more likely to die, (Table 2). Antenatal and delivery characteristics that were associated with increased risk of death included: inadequate antenatal care, operative or assisted vaginal deliveries, obstructed labor, fetal malposition, antepartum hemorrhage, hypertensive disorders, and the receipt of many maternal medical treatments (e.g: blood transfusions, anticonvulsant medications, intravenous fluids), (Table 3). Postpartum characteristics that were associated with maternal death included seizures or convulsions, hemorrhage, anemia, hospitalization and the receipt of medical treatment, (Table 4).

**Table 2:** Maternal characteristics by maternal status at 6 weeks

Characteristic	n(%)^*^			RR of maternal death versus alive at six weeks (95% CI) ^‡^
		
	Women who died n=402	Women alive six weeks after delivery n=277,334	Total n=277,736	
Maternal age				

< 20	36 (9.0)	32,987 (11.9)	33,023 (11.9)	0.8 (0.6, 1.1)

20-35	332 (83.4)	233,322 (84.3)	233,654 (84.3)	1.0

> 35	30 (7.5)	10,594 (3.8)	10,624 (3.8)	2.0 (1.3, 3.0)

Maternal education				

No formal education	164 (41.1)	69,204 (25.1)	69,368 (25.1)	4.2 (2.2, 8.0)

Primary	132 (33.1)	103,663 (37.5)	103,795 (37.5)	2.3 (1.2, 4.6)

Secondary	92 (23.1)	83,276 (30.2)	83,368 (30.2)	2.0 (1.0, 4.0)

University +	11 (2.8)	19,953 (7.2)	19,964 (7.2)	1.0

Parity				

0	131 (32.7)	93,337 (33.8)	93,468 (33.8)	1.3 (1.0, 1.6)

1-2	131 (32.7)	117,115 (42.4)	117,246 (42.3)	1.0

> 2	139 (34.7)	66,071 (23.9)	66,210 (23.9)	1.8 (1.4, 2.2)

Last pregnancy did not result in a live birth	34 (12.6)	11,132 (6.1)	11,166 (6.1)	2.1 (1.5, 3.1)

^*^ The denominator used to determine the percentage of women with each characteristic varies due to missing data.

^‡^ Generalized linear models were used to evaluate the relationship of potential factors and maternal mortality and to develop point and interval estimates of relative risk associated with these factors. Generalized estimating equations were used to account for the correlation of outcomes within cluster to develop appropriate confidence intervals. Unless otherwise noted, the reference group is women who did not have the listed characteristic.

**Table 3:** Antenatal and delivery characteristics by maternal status at 6 weeks

Characteristic	n(%)^*^			RR of maternal death versus alive at six weeks (95% CI) ^‡^
	Women who died n=402	Women alive six weeks after delivery n=277,334	Total n=277,736	
		
No antenatal care	29 (7.4)	10,640 (3.8)	10,669 (3.9)	1.7 (1.2, 2.5)

Fewer than 4 antenatal care visits	94 (59.1)	72,527 (48.4)	72,621 (48.4)	1.5 (1.1, 2.2)

*Birth attendant*				

Physician	148 (46.8)	106,671 (38.5)	106,819 (38.5)	1.0

Nurse/midwife/health worker	69 (21.8)	88,243 (31.8)	88,312 (31.8)	0.5 (0.4, 0.7)

Traditional birth attendant	66 (20.9)	63,817 (23.0)	63,883 (23.0)	0.6 (0.4, 0.8)

Family/other	33 (10.4)	18,558 (6.7)	18,591 (6.7)	1.1 (0.8, 1.7)

*Delivery location*				

Hospital	169 (53.3)	126,426 (45.6)	126,595 (45.6)	1.0

Clinic	52 (16.4)	68,629 (24.8)	68,681 (24.7)	0.5 (0.4, 0.7)

Home/other	96 (30.3)	82,197 (29.6)	82,293 (29.6)	0.7 (0.6, 1.0)

*Delivery mode*				

Vaginal	222 (71.4)	230,853 (86.1)	231,075 (86.1)	1.0

Vaginal assisted	15 (4.8)	4,145 (1.5)	4,160 (1.6)	3.4 (1.8, 6.6)

Caesarean section	74 (23.8)	32,973 (12.3)	33,047 (12.3)	2.4 (1.8, 3.2)

Obstructed labor	79 (20.4)	29,735 (10.7)	29,814 (10.8)	2.0 (1.6, 2.6)

Fetal Malposition	27 (7.1)	5,879 (2.1)	5,906 (2.1)	3.4 (2.3, 5.0)

Antepartum hemorrhage	46 (11.9)	6,089 (2.2)	6,135 (2.2)	5.7 (3.7, 8.8)

Hypertensive disorders	78 (20.5)	8,039 (2.9)	8,117 (2.9)	8.4 (6.3, 11.2)

*Maternal treatment provided*				

Antibiotics	173 (52.9)	103,941 (37.7)	104,114 (37.8)	2.1 (1.7, 2.7)

Corticosteroids	12 (9.4)	6,060 (5.3)	6,072 (5.3)	1.8 (0.9, 3.5)

Oxytocics (including misoprostol)	140 (43.3)	108,524 (39.5)	108,664 (39.5)	1.2 (1.0, 1.6)

Blood transfusion	92 (28.3)	3,889 (1.4)	3,981 (1.4)	26.6 (19.7, 36.0)

Removal of retained products	42 (12.9)	15,799 (5.7)	15,841 (5.7)	2.7 (1.7, 4.3)

Anticonvulsants	31 (9.7)	3,288 (1.2)	3,319 (1.2)	8.9 (5.6, 14.0)

IV Fluids	188 (57.5)	93,590 (33.9)	93,778 (34.0)	2.9 (2.2, 3.7)

Forceps/vacuum	13 (4.0)	1,913 (0.7)	1,926 (0.7)	5.6 (3.1, 10.0)

Other surgery/treatment	24 (7.4)	10,891 (4.0)	10,915 (4.0)	2.1 (1.3, 3.3)

^*^ The denominator used to determine the percentage of women with each characteristic varies due to missing data.

^‡^ Generalized linear models were used to evaluate the relationship of potential factors and maternal mortality and to develop point and interval estimates of relative risk associated with these factors. Generalized estimating equations were used to account for the correlation of outcomes within cluster to develop appropriate confidence intervals. Unless otherwise noted, the reference group is women who did not have the listed characteristic.

**Table 4:** Postpartum maternal characteristics by maternal status at 6 weeks

Characteristic	n(%)^*^			RR of maternal death versus alive at six weeks (95% CI) ^‡^
		
	Women who died n=402	Women alive six weeks after delivery n=277,334	Total n=277,736	
Seizures/convulsions	32 (23.9)	227 (0.1)	259 (0.1)	325.3 (212.4, 498.0)

Hemorrhage	27 (20.5)	1,491 (0.5)	1,518 (0.6)	45.3 (25.6, 80.1)

Anemia	48 (36.1)	5,304 (1.9)	5,352 (1.9)	28.5 (17.4, 46.8)

Hospitalized	53 (35.6)	750 (0.3)	803 (0.3)	185.7 (125.5, 274.8)

Received medical treatment	88 (59.1)	9,803 (3.6)	9,891 (3.6)	38.1 (24.6, 59.0)

^*^ The denominator used to determine the percentage of women with each characteristic varies due to missing data.

^‡^ Generalized linear models were used to evaluate the relationship of potential factors and maternal mortality and to develop point and interval estimates of relative risk associated with these factors. Generalized estimating equations were used to account for the correlation of outcomes within cluster to develop appropriate confidence intervals. Unless otherwise noted, the reference group is women who did not have the listed characteristic.

**Figure 2:**
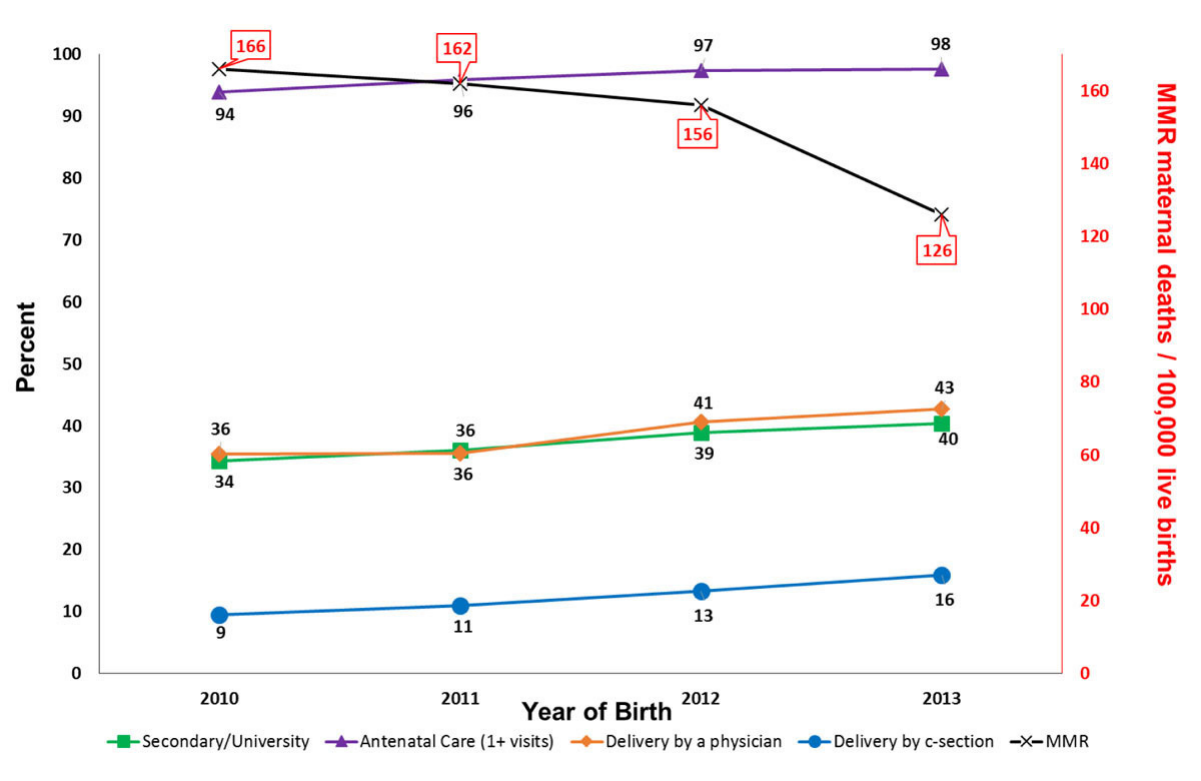
Social factors and measures of care, by year^*^ MNHR 2010-2013 deliveries excluding women lost to follow-up prior to delivery or missing maternal status at 6 weeks after delivery.

**Figure 3:**
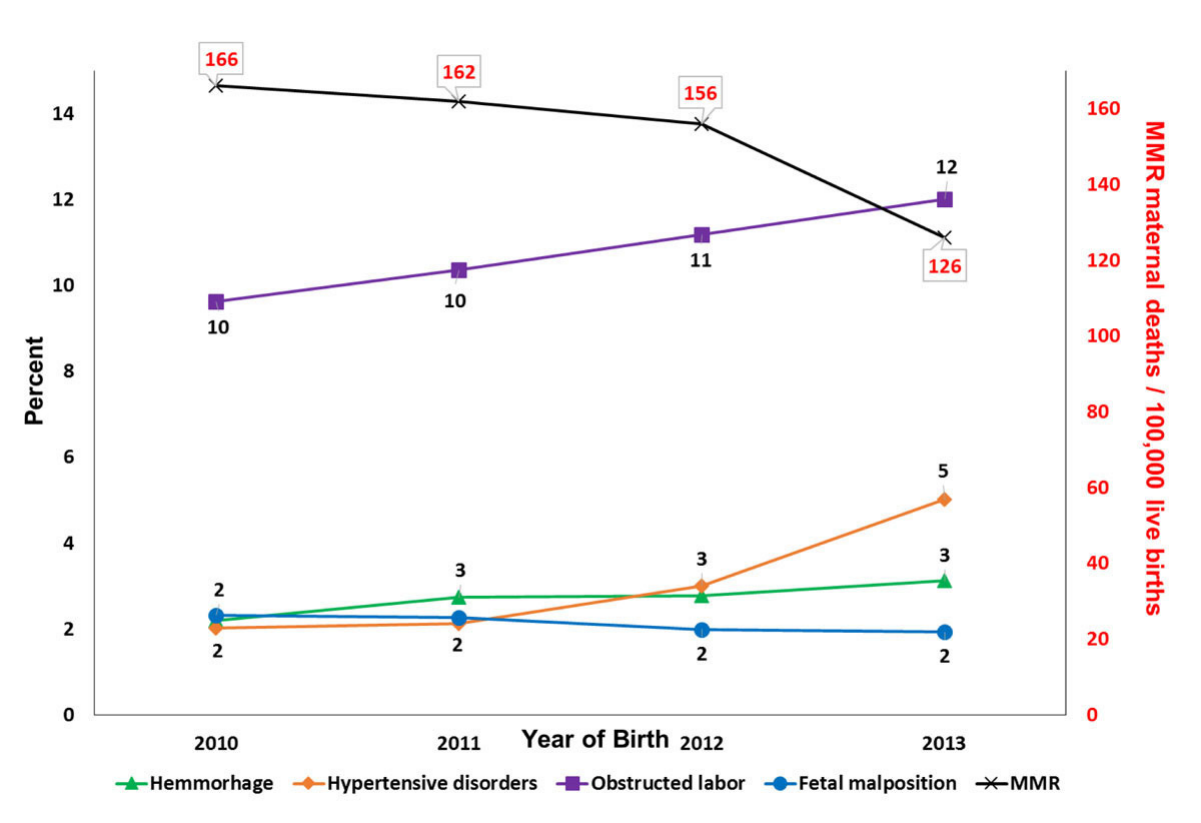
Pregnancy complications, by year^*^ MNHR 2010-2013 deliveries excluding women lost to follow-up prior to delivery or missing maternal status at 6 weeks after delivery.

We evaluated the association between selected factors that were associated with the death of the mother, (Table 5). We excluded the factor of a previous living child due to missing data or primiparous mothers (n=94,393). We excluded the variable of delivery location due to significant confounding with birth attendant. When adjusted for study site and the other variables, no formal education (RR 3.2 [1.5, 6.9]), primary education only (RR 3.4 [1.6, 7.5]), secondary education only (RR 2.5 [1.1, 5.7]), lack of antenatal care (RR 1.8 [1.2, 2.5]), caesarean section delivery (RR 1.9 [1.3, 2.8]), hemorrhage (RR 3.3 [2.2, 5.1]), and hypertensive disorders (RR 7.4 [5.2, 10.4]) were associated with a higher risk of death. Using the variables that were found to be significant in the model, we evaluated social factors and measures of care that might influence MMR by year (Figure 2) and pregnancy complications that might influence MMR by year (Figure 3).

**Table 5:** Multivariable model with outcome of maternal death by 6 weeks after delivery^*^

Model Covariates	Adjusted Relative Risk
Maternal age	

Maternal age: <20 vs. 20-35	1.1 (0.7, 1.6)

Maternal age: >35 vs. 20-35	1.2 (0.6, 2.4)

Maternal education	

Maternal education: no formal vs university	3.2 (1.5, 6.9)

Maternal education: primary vs university	3.4 (1.6, 7.5)

Maternal education: secondary vs university	2.5 (1.1, 5.7)

Parity	

Parity:0 vs. 1-2	1.3 (1.0, 1.7)

Parity:>2 vs. 1-2	1.4 (1.0, 2.0)

No antenatal care vs. at least 1 antenatal care visit	1.8 (1.2, 2.5)

Birth attendant	

Birth attendant: nurse/midwife/health worker vs physician	0.4 (0.3, 0.6)

Birth attendant: traditional birth attendant vs physician	0.6 (0.3, 1.0)

Birth attendant: family/other vs physician	0.4 (0.2, 0.7)

Delivery mode	

Delivery mode: vaginal-assisted vs vaginal	1.8 (0.9, 3.7)

Delivery mode: caesarean-section vs vaginal	1.9 (1.3, 2.8)

Obstructed labor: yes vs no	1.1 (0.8, 1.5)

Fetal Malposition: yes vs no	1.3 (0.8, 2.0)

Hemorrhage: yes vs. no	3.3 (2.2, 5.1)

Hypertensive disorders including seizures / convulsions: yes vs. no	7.4 (5.2, 10.4)

^*^ MNHR 2010-2013 deliveries excluding women lost to follow-up prior to delivery or missing maternal status at 6 weeks after delivery. Multivariable model adjusted for clinical site.

## Discussion

Sites in our population-based multi-national registry had a combined MMR of 153, a number which is higher than the WHO global target for 2030 of less than 70 maternal deaths per 100,000 live births and higher than the supplementary goal that no country should have an MMR greater than 140 [14]. This observation highlights the continued challenge of maternal mortality and the need to improve the health and medical care of pregnant women in some areas. We recorded considerable variation in the MMR by site with a range of 72 in Argentina to 321 in Pakistan. Although, we did not observe a consistent yearly decline in MMR in each site every year, our overall MMR for all sites did improve, except for Pakistan. Pakistan was an outlier with the highest MMR compared to all other sites each year with no improvement over 4 years. The potential reasons for the high MMR in the Pakistan site are addressed elsewhere [15].

We performed exploratory analyses to investigate the association with positive and negative factors that might have contributed to the overall decline in MMR. We observed an increase in the percentage of deliveries that were attended by a physician and delivery by caesarean section, but it is not clear that the change in either of these factors alone explains the decline in MMR. We did not observe a decrease in complications of pregnancy (fetal malposition, obstructed labor, hemorrhage or hypertensive disorders) that might have explained the lower MMR. In fact, some of these underlying risk factors increased over time. Taken together, these observations might indicate that improvements in measures of care, without a decline in underlying disease prevalence improve maternal mortality rates. Another possibility is our study sites include participants potentially enrolled in various research studies to improve maternal and newborn health. Although we did not observe a consistent improvement in maternal mortality in each site every year, it is possible that participation in our research studies has decreased overall maternal mortality.

We identified important modifiable factors associated with maternal death. Consistent with previously published works, we identified hemorrhage and hypertensive disorders as treatable factors that are the most highly associated with maternal death [16-18]. We also identified an increased risk of death among women whose deliveries were attended by a physician and those who had a caesarean section. These associations may result from confounding by the indication for the operative delivery, however the indications for these deliveries were not collected in this dataset. We presume that women are delivered by caesarean section in response to a condition that places them in a higher risk category for mortality; therefore these women have a higher baseline risk of mortality. This presumption is supported by previous publications that report that the largest proportion of maternal deaths take place in health care facilities where underlying conditions that often result in mortality are more likely to be treated [15].

The MNHR has many strengths for analyses of factors for maternal death. We have a large data set of pregnancies and delivery outcomes in low and middle-income countries that has been prospectively collected and validated. Most multi-country data from low and middle-income countries are not of such high-quality and many rely on model-based estimates for maternal mortality. We include all pregnant women who reside within study community, therefore providing population-level data for each region represented. We enroll women who deliver in facilities and outside of facilities and do not rely on the health systems in order to identify eligible women, therefore representing a cross-sectional cohort of residents within these regions. We have an extremely high follow-up rate, which permits complete data to 6 weeks post-partum.

Despite our robust, prospective data set, our study design limits generalizability to other low- and middle-income countries. Although we were able to capture nearly all of the pregnancies within the study communities, our communities might not reflect delivery outcomes in diverse regions within each country. Our study sites generally cover one district or province that might have different birth outcomes compared to other regions within the country, limiting our ability to draw inferences about mortality within each country as a whole. Although our goal is to identify women early in pregnancy, sites varied in their ability to identify pregnancies early, therefore it is possible that we have under-reported maternal deaths occurring early in pregnancy or those related to medical termination of pregnancy or miscarriage, as these pregnancies represented only 3.4% of our sample. Although robust, our dataset does not record all factors that might be associated with maternal mortality, such as: maternal nutritional status, timing of referral, or access to medical care. Our data collection techniques limited our ability to assign an accurate cause of death. For example, we collected data on the presence of sepsis around the time of delivery, but there was significant variability in reporting by site, therefore we have excluded this variable from our report. Our team has developed a cause of death algorithm to address this problem and will be testing this tool in the future [19].

The MNHR can be used to identify preventable causes of maternal mortality in diverse settings. The MNHR has utility in informing public health strategies that can be monitored over time to determine their impact on reducing maternal mortality. Systematic data collection strategies that include all births in a region are essential to understanding the underlying causes of maternal mortality and should guide the development of comprehensive intervention strategies to reduce maternal mortality.

## Competing interests

The authors declare they have no competing interests.

## Authors’ contributions

MB and CB conceived of the study analyses and MB wrote the first draft with input from CB, AL, VT and EMM. AL, AT, SSG, FE, AG, SS, OP, AP, JB, BK, EC, EAL, KKM, NFK, RJD, PLH, WAC, MKT, RLG, EMM, and CLB developed the initial study protocol. AL, AM, AP, FA, AG, SS, SSG, and FE oversaw field activities and monitoring. VT and DDW performed the statistical analyses. All authors read and approved the final manuscript.

## Peer review

Reviewer reports for this article can be found in Additional file 1.

## Supplementary Material

Additional file 1 Click here for file

## Declarations

This article has been published as part of *Reproductive Health* Volume 12 Supplement 2, 2015: Research reports from the NICHD Global Network for Women’s and Children’s Health Research Maternal and Newborn Health Registry. The full contents of the supplement are available online at http://www.reproductive-health-journal.com/supplements/12/S2. Publication of this supplement was supported by grants from the Eunice Kennedy Shriver National Institute of Child Health and Human Development to RTI International.
