# Supplementary material for: Risk factors for maternal death and trends in maternal mortality in low- and middle-income countries: a prospective longitudinal cohort analysis
Source: Reprod Health. 2015 Jun 8;12(Suppl 2):S5. doi: 10.1186/1742-4755-12-S2-S5 (PMC4464034; doi:10.1186/1742-4755-12-S2-S5)
Supplement: Additional file 1 [file 1742-4755-12-S2-S5-S1.pdf]

Referee's comments to the authors– this sheet WILL be seen by the author(s) and published with the article

Title	Risk factors for maternal death in low- and middle-income countries: a prospective longitudinal cohort analysis
Author(s)	Melissa Bauserman, Adrien Lokangaka, Vanessa Thorsten, Antoinette Tshefu, Shivaprasad S Goudar, Fabian Esamai, Ana Garces, Sarah Saleem, Omrana Pasha, Archana Patel, Elwyn Chomba, Bhala Kodkany, Edward A Liechty, K Michael Hambidge, Nancy F Krebs, Richard J Derman, Patricia L Hibberd, Albert Manasyan, Fernando Althabe, Waldemar A Carlo, Marion Koso-Thomas, Robert L Goldenberg, Dennis D Wallace, Elizabeth M McClure, and Carl L Bose
Referee's name	Cheryl Moyer

When assessing the work, please consider the following points, where applicable:

1. Is the question posed by the authors new and well defined?
2. Are the methods appropriate and well described, and are sufficient details provided to replicate the work?
3. Are the data sound and well controlled?
4. Does the manuscript adhere to the relevant standards for reporting and data deposition?
5. Are the discussion and conclusions well balanced and adequately supported by the data?
6. Do the title and abstract accurately convey what has been found?
7. Is the writing acceptable?

Please make your report as constructive and detailed as possible in your comments so that authors have the opportunity to overcome any serious deficiencies that you find and please also divide your comments into the following categories:

- Major Compulsory Revisions (which the author must respond to before a decision on publication can be reached)
- Minor Essential Revisions (such as missing labels on figures, or the wrong use of a term, which the author can be trusted to correct)
- Discretionary Revisions (which are recommendations for improvement but which the author can choose to ignore)

Where possible please supply references to substantiate your comments.

When referring to the manuscript please provide specific page and paragraph citations where appropriate.

General comments:
The authors have presented analysis of multi-year, multi-country data regarding risk factors for maternal mortality in six countries. Overall, the manuscript is thorough, the methods are sound, and the results worthy of publication. My specific comments are as follows:

Minor revisions:
The abstract would benefit from mention of the variability in MMR across Africa, Asia, and Latin America.

In the middle of Page 7, the authors speculate that women delivered by a physician or with an operative delivery have a higher baseline risk of mortality and are therefore more likely to be delivered at a health facility under a physician's care. This statement seems to be hinting at the bigger issue here that isn't quite clear in the authors' discussion: two of the biggest risk factors found to be associated with an increased likelihood of death seem less like 'risk factors' and more like responses to potentially poor prognoses / attempts to address potentially bad outcomes. A c-section is probably not best characterized as a risk factor for death – it's whatever the underlying condition is that warrants a c-section that is more likely to be the 'risk factor'.

(continue on the next sheet)

Continued:

The manuscript would be improved if the authors could address this a bit more carefully – even though I am sure that space constraints may make such a nuanced discussion difficult.

While the manuscript describes a very robust mechanism for identifying risk factors for maternal mortality, and for identifying preventable causes of maternal mortality, I would like to have seen a better developed discussion about the “so what” factor of identifying education, ANC, c-sections, hemorrhage, and hypertensive disorders as the leading causes of maternal mortality. This does not seem to be particularly new information. What is the benefit of seeing this across 6 sites? And what are the implications?

Referee's comments to the authors– this sheet WILL be seen by the author(s) and published with the article

Title	Risk factors for maternal death in low- and middle-income countries: a prospective longitudinal cohort analysis
Author(s)	Melissa Bauserman, Adrien Lokangaka, Vanessa Thorsten, Antoinette Tshefu, Shivaprasad S Goudar, Fabian Esamai, Ana Garces, Sarah Saleem, Omrana Pasha, Archana Patel, Elwyn Chomba, Bhala Kodkany, Edward A Liechty, K Michael Hambidge, Nancy F Krebs, Richard J Derman, Patricia L Hibberd, Albert Manasyan, Fernando Althabe, Waldemar A Carlo, Marion Koso-Thomas, Robert L Goldenberg, Dennis D Wallace, Elizabeth M McClure, and Carl L Bose
Referee's name	Marshall Carpenter

When assessing the work, please consider the following points, where applicable:

1. Is the question posed by the authors new and well defined? Yes
2. Are the methods appropriate and well described, and are sufficient details provided to replicate the work?
3. Are the data sound and well controlled? No
4. Does the manuscript adhere to the relevant standards for reporting and data deposition? Unknown
5. Are the discussion and conclusions well balanced and adequately supported by the data? No
6. Do the title and abstract accurately convey what has been found? Yes
7. Is the writing acceptable? Yes

Please make your report as constructive and detailed as possible in your comments so that authors have the opportunity to overcome any serious deficiencies that you find and please also divide your comments into the following categories:

- Major Compulsory Revisions (which the author must respond to before a decision on publication can be reached)
- Minor Essential Revisions (such as missing labels on figures, or the wrong use of a term, which the author can be trusted to correct)
- Discretionary Revisions (which are recommendations for improvement but which the author can choose to ignore)

Where possible please supply references to substantiate your comments.

When referring to the manuscript please provide specific page and paragraph citations where appropriate.

General comments: See below

Major compulsory revisions:

Minor essential revisions:

Discretionary revisions:

(continue on the next sheet)

Continued:
Since this is a prospectively derived data set, this reviewer assumes that additional information is available regarding events leading to maternal death beyond that analyzed in this report. The report of this very large cohort finds associations with odds ratios above 5 for only antepartum hemorrhage and hypertension. Aside from less robust associations found with abnormal fetal lie or obstructed labor, these are the only associations of maternal death with causative factors. Most other associations identify conditions (eg educational attainment) that are not directly related to abnormal pregnancy condtions or treatments of morbid pregnancy rather than causes of maternal morbidity or death. Since the rationale for the study was identification of risk factors that, when identified early, would lead to development of "comprehensive intervention strategies to preven pregnancy-related complications, this report, failing to examine available data to determine plausible causative factors associated with maternal death does not meet its goal.

Lesser criticisms include:

1. Although the WHO maternal death criteria include those associated with "miscarriage," the certainly poor identification of these events and those of induced abortion confounds comparisons among research sites and across time. Additional analyses based on statistics of maternal deaths after 20 gestational weeks (for example) or other criteria that will allow more secure and stable ascertainment should also be reported.
2. Neither the Bose et al original study description, nor this manuscript provide any data allowing insight as to the success of each of the study venues in identifying gravidas nor quantify the type and frequency of problems establishing and maintaining subject cohorts. The data provided in figure 1 are tantalizing, but the reader is left with no insight as to differences in case ascertainment or causative factors that set the Pakistan venue apart from the others.
3. Sepsis is likely a significant cause of maternal death, especially in venues where obstructed labor treatment is delayed. Have the authors examined surrogate variables that may identify septic patients?

Supplement Editor comments:

Dear authors,
As you can see the reviewers raised serious concerns about your manuscript. Our decision was to give you a chance to reply to such concerns and the possibility of a re-review of your manuscript.
In addition to these comments please take in account that the manuscript should be read and understood without the need to go over other articles. In this regard I consider that a better description of the population could be of help. In the Introduction your message is about the lack of similar information than the one of your paper without giving a systematic review about this lack. My advice would be to write these statements as a positive message like the need and advantages to make contributions in this subject.
Please in your reply provide us:
1) one "clean" copy of your manuscript
2) one copy where your changes are highlighted (tracked changes).
3) A separate, point by point response to the editor and referee comments.
